# IL-29 enhances Toll-like receptor-mediated IL-6 and IL-8 production by the synovial fibroblasts from rheumatoid arthritis patients

**DOI:** 10.1186/ar4357

**Published:** 2013-10-29

**Authors:** Lingxiao Xu, Xiaoke Feng, Wenfeng Tan, Weijuan Gu, Dunming Guo, Miaojia Zhang, Fang Wang

**Affiliations:** 1 Department of Rheumatology, The First Affiliated Hospital of Nanjing Medical University, 300 Guangzhou Road, Nanjing, Jiangsu Province 210029, China; 2 Department of Cardiology, The First Affiliated Hospital of Nanjing Medical University, 300 Guangzhou Road, Nanjing, Jiangsu Province 210029, China; 3 Department of Orthopaedics, The First Affiliated Hospital of Nanjing Medical University, 300 Guangzhou Road, Nanjing, Jiangsu Province 210029, China

## Abstract

**Introduction:**

We previously reported that IL-29, a newly described member of interferon (IFN) family, was overexpressed in blood and synovium of rheumatoid arthritis (RA) patients and triggered proinflammatory cytokine IL-6 and IL-8 mRNA expression in RA synovial fibroblasts (RA-FLS). This suggests that IL-29 has an important role in synovial inflammation. Toll-like receptors (TLRs) also activate RA-FLS to produce inflammatory mediators including tumor necrosis factor α (TNF-α) and IL-1β in RA-FLS. Since the TLR family plays an early role in the innate immune response and the subsequent induction of the adaptive immune response, we hypothesize that IL-29 interacts with TLRs in RA inflammation. This study aimed to investigate the effect of IL-29 on TLR-mediated proinflammatory cytokine production in RA-FLS.

**Methods:**

The mRNA level of IL-29 receptors (IL-28Rα and IL-10R2) in RA-FLS was determined by semi-quantitative RT- PCR. IL-6 and IL-8 mRNA expressions in RA-FLS were evaluated by real-time PCR after pre-incubation with IL-29 and subsequent stimulation with peptidoglycan (PGN, TLR2 ligand), or polycytidylic acid (poly(I:C), TLR3 ligand), or lipopolysaccharide (LPS, TLR4 ligand) . The production of TLR2, 3, and 4 in RA-FLS after IL-29 stimulation was also assessed by real-time PCR and flow cytometry. IL-29 mRNA and protein expression in RA-FLS after stimulation with PGN, poly(I:C), or LPS were measured by real-time PCR and enzyme-linked immunosorbent assay (ELISA), respectively.

**Results:**

The IL-29 receptor complex (IL-28Rα and IL-10R2) was identified in RA-FLS. IL-29 enhanced TLR-mediated IL-6 and IL-8 expression in RA-FLS. IL-29 upregulated expression of TLR2, 3 and 4 in RA-FLS. Exposure to PGN, poly(I:C) or LPS triggered IL-29 production by RA-FLS.

**Conclusions:**

We show for the first time that IL-29 enhances TLR-induced proinflammatory cytokine production in RA-FLS via upregulation of TLRs.

## Introduction

The classical IFN family cytokines, such as types I and II IFNs, are known to play an important role in both innate and adaptive immune responses during viral infection and autoimmune inflammation. Type III IFNs, or IFN-λs, are a newly described member of the IFN family, including IFN-λ1 (IL-29), IFN-λ2 (IL-28A) and IFN-λ3 (IL-28B). Each member of type III IFNs signals through the heterodimeric receptor complex, including the IFN-λR (IL-28Rα) chain and IL-10 receptor type 2 (IL-10R2) chain [1,2]. IL-28Rα expression is cell-specific, whereas IL-10R2 is broadly expressed in most cell types, such as type I IFN receptors [2]. Type III IFNs bind to their receptors and activate the Janus kinase/signal transducers and activators of transcription (JAK-STAT), mitogen-activated protein kinase and Akt signaling pathways to induce antiviral, antiproliferative, antitumor and immune responses [3-5]. Although the antiviral activity has been studied extensively during the past decade, the immunoregulatory role of type III IFNs in immune cells is still poorly understood.

The evidence for an immunoregulatory role of IL-29 is derived mostly from *in vitro* studies. For example, IL-29 inhibits the release of IL-13 in T cells [6,7]; increases the production of IL-6, IL-8 and IL-10 in macrophages [8]; and induces the secretion of IL-6 and TNF from CD14^+^ T cells [9] as well as IL-4 and IL-13 from mast cells [10]. Investigators in a recent study reported that IL-29 induces the secretion of chemokines IFN-γ-inducible protein 10, monokine induced by IFN-γ and IL-8 in peripheral blood mononuclear cells (PBMCs) [11,12] in patients with systemic lupus erythematosus (SLE), implying its involvement in SLE pathogenesis. Significantly, our recent study was the first showing that IL-29 was highly expressed in PBMCs, serum, synovial fluid and synovium in rheumatoid arthritis (RA) patients [13]. However, little is known about what triggers the expression of IL-29 and how elevated IL-29 levels drive synovial inflammation during RA development.

It has recently been postulated that the interaction of genetic and environmental factors contributes to RA pathogenesis. For instance, Toll-like receptors (TLRs) may recognize cytomegalovirus [14,15] and Epstein-Barr virus [16] and trigger autoimmunity in the host. TLRs are phylogenetically conserved molecules that recognize pathogen-associated molecular patterns and eliminate invading pathogens by activation of immune responses. In RA, several TLRs, including TLR2, TLR3, TLR4 and TLR7, are highly expressed in the synovial tissues [17], and the ligands of TLRs induce the expression of cytokines (IL-1β, IL-6 and IFN-α), chemokines (IL-8) and matrix metalloproteinases on synovial fibroblasts (FLSs) [18-21], which contributes to RA synovial inflammation. Interestingly, proinflammatory cytokines such as IL-1β itself further upregulate TLR expression on FLSs [22,23], suggesting autoamplification of inflammation in RA-FLS through interaction of TLRs and cytokines.

This notion is further supported by the evidence that IFNs are able to modulate TLR-mediated cytokine production in immune cells. It has been shown that IFN-α enhances the expression of TLR3 and TLR7, leading to increased production of IL-6 and TNF-α in RA-FLS [24]. In addition, TLR4- and TLR8-induced p40 subunit of IL-12 (IL-12p40) production is increased by IL-29 in human monocyte-derived macrophage [25]. Despite this evidence, whether IL-29 is also involved in TLR-induced proinflammatory cytokine production in RA-FLS has not been reported. Considering that both IL-29 and TLRs could elevate mRNA levels of IL-6 and IL-8 in RA-FLS, we examined the effect of IL-29 on TLR-mediated IL-6 and IL-8 production contributing to RA inflammation.

In the present study, we show for the first time that TLR2, TLR3 and TLR4 engagement on RA-FLS induces endogenous IL-29, which further enhances IL-6 and IL-8 expression by upregulation of TLR2, TLR3 and TLR4 expression. Our findings demonstrate that, in addition to its potent antiviral activity, IL-29 plays an important role in modulating cytokine production in RA-FLS, which may enhance immune responses to the pathogens contributing to RA inflammation.

## Methods

### Reagents

TRIzol reagent and SYBR Green I stain were purchased from Invitrogen (Carlsbad, CA, USA). ExScript RT reagent kit and SYBR *Premix Ex Taq* were obtained from TaKaRa (Dalian, China). Human IL-29 ELISA kits were purchased from Adlitteram Diagnostic Laboratories (San Diego, CA, USA), human IL-6 and IL-8 ELISA kits were obtained from ExCell Biology Inc (Shanghai, China). Cell proliferation reagent WST-1 was purchased from Roche Diagnostics GmbH (Roche Applied Science, Mannheim, Germany). A cell fixation permeabilization kit and Cytofix/Cytoperm solution were purchased from BD Pharmingen (San Jose, CA, USA), fluorescein isothiocyanate (FITC)-conjugated anti-human TLR2 (clone TL2.1), phycoerythrin-conjugated anti-human TLR3 (clone TLR3.7) and TLR 4 (clone HTA125) monoclonal antibody or isotope control were obtained from eBioscience (San Diego, CA, USA). DMEM and FBS were purchased from Gibco (Carlsbad, CA, USA). Recombinant human IL-29 and TNF-α were purchased from Peprotech (Rocky Hill, NJ, USA). Polyinosinic-polycytidylic acid (poly(I:C)) and peptidoglycan (PGN) were obtained from Invivogen (San Diego, CA, USA). Lipopolysaccharide (LPS) was purchased from Sigma-Aldrich (St Louis, MO, USA).

### Cell culture

Human rheumatoid fibroblast-like synoviocyte MH7A cells were isolated from intra-articular soft tissue of the knee joints of RA patients and were established as a cell line by transfection with the Simian virus 40 T antigen [26]. The cell line was kindly provided by Dr David Yu (University of California, Los Angeles, CA, USA), and ethical approval for their use was waived by the Review Board of the First Affiliated Hospital of Nanjing Medical University. MH7A cells were cultured in DMEM supplemented with 10% FBS, 100 U/ml penicillin and 100 μg/ml streptomycin at 37°C in a humidified atmosphere of 5% CO_2_ in air. Details regarding primary RA fibroblast isolation and culture can be found in Additional file 1.

### Stimulation of synovial fibroblasts

To study functional upregulation of IL-29, we stimulated MH7A cells with various concentrations of IL-29 (1, 10 and 100 ng/ml) or TNF-α (10 ng/ml) for 24 hours. After incubation, the cell supernatants were collected for analysis of IL-6 and IL-8 production measured by ELISA as described below.

To study functional upregulation of TLR2, TLR3 and TLR4, we cultured MH7A cells with IL-29 (100 ng/ml) for 24 hours and subsequently stimulated cells with the TLR2, TLR3 and TLR4 ligand PGN (2.5 μg/ml), poly(I:C) (25 μg/ml) or LPS (100 ng/ml), respectively. After a further 24 hours, MH7A cells were collected for analysis of IL-6 and IL-8 expression by real-time PCR.

To study TLR expression, we provoked MH7A cells with various concentrations of IL-29 (1, 10 and 100 ng/ml) or TNF-α (10 ng/ml; positive control) for 24 hours. After incubation, MH7A cells were collected for analysis of TLR2, TLR3 and TLR4 expression by real-time PCR and flow cytometry assay.

To study IL-29 expression, we stimulated MH7A cells with TLR2, TLR3 or TLR4 ligand PGN (2.5 μg/ml), poly(I:C) (25 μg/ml) or LPS (100 ng/ml) for 24 hours, respectively. After incubation, the cells were collected for analysis of IL-29 gene expression by real-time PCR and analysis of cell supernatants for IL-29 protein expression by ELISA.

### Viability assay by MTT

Cell viability was assessed by 3-(4,5-dimethylthiazol-2-yl)-2,5-diphenyltetrazolium bromide (MTT) assay after MH7A cell incubation with IL-29. Briefly, MH7A cells (5 × 10^3^) were incubated in a 96-well, flat-bottomed culture plate in a final volume of 200 μl/well culture medium with or without IL-29 (100 ng/ml) for 24 hours in a humidified atmosphere (37°C at 5% CO_2_). Next, 20 μl of cell proliferation reagent WST-1 were added to each well and incubated for a further 4 hours. After incubation, the plates were shaken thoroughly for 1 minute, and the absorbance was measured at 450 nm and 630 nm using an ELISA plate reader (BioTek, Winooski, VT, USA).

### Semiquantitative RT-PCR and quantitative real-time PCR analysis

The mRNA expression of the IL-29 complex, receptor IL-28Rα and IL-10R2, in MH7A cells was determined by semiquantitative PCR. Total RNA from the cells was isolated using TRIzol reagent. The cDNA was prepared using the ExScript RT reagent kit according to the manufacturer’s instructions and then was amplified by PCR with specific primers for human IL-28Rα (5′-ACCTATTTTGTGGCCTATCAGAGCT-3′ and 5′-CGGCTCCACTTCAAAAAGGTAAT-3′), IL-10R2 (5′-GGCTGAATTTGCAGATGAGCA-3′ and 5′-GAAGACCGAGGCCATGAGG-3′), glyceraldehyde 3-phosphate dehydrogenase (GAPDH) (5′-AGAAGGCTGGGGCTCATTTG-3′ and 5′-AGGGGCCATCCACAGTCTTC-3′). The conditions for each amplification were as follows: 95°C for 45 seconds, 40 cycles of denaturation at 95°C for 30 seconds, annealing temperatures at 59°C for 30 seconds and extension at 72°C for 35 seconds. The PCR products were electrophoresed on 2.0% agarose gels, stained with SYBR Green I Nucleic Acid Gel Stain (Invitrogen) and visualized by using a gel imaging system (ChemiDoc XRS + System; Bio-Rad Laboratories, Hercules, CA, USA).

The mRNA of IL-29, IL-6, IL-8, TLR2, TLR3 and TLR4 in MH7A cells was determined by real-time PCR. Briefly, after synthesizing cDNA from the total RNA by using the ExScript RT reagent kit, real-time PCR was performed using SYBR *Premix Ex Taq* on a sequence detection system (Eppendorf, Hamburg, Germany). Each reaction contained 12.5 μl of 2× SYBR Green Master Mix, 1 μl of 10 μM primers and 1 μl of cDNA to a total volume of 20 μl. The thermal cycling conditions included an initial denaturation step at 95°C for 30 seconds, followed by 40 cycles of 5 seconds at 95°C and 30 seconds at 60°C. Primer sequences for human IL-29, IL-6, IL-8, TLR2, TLR3, TLR4 and GAPDH are summarized in Table 1. Relative gene expression was determined by the 2^-ΔΔCt^ method.

**Table 1:** Gene primers for quantitative PCR analysis

**Gene name**	**Forward (5′ to 3′)**	**Reverse (5′ to 3′)**
Human *IL-29*	GAAGCAGTTGCGATTTAGCC	GAAGCTCGCTAGCTCCTGTG
Human *IL-6*	AACCTGAACCTTCCAAAGATGG	TCTGGCTTGTTCCTCACTACT
Human *IL-8*	CATACTCCAAACCTTTCCACCCC	TCAGCCCTCTTCAAAAACTTCTCCA
Human *TLR2*	TGTTGCAAGCAGGATCCAAAG	CACAAAGTATGTGGCATTGTCCAG
Human *TLR3*	GGACTTTGAGGCGGGTGTT	TGTTGAACTGCATGATGTAC CTTGA
Human *TLR4*	AGGATGATGCCAGCATGATGTC	TCAGGTCCAGGTTCTTGGTTGAG
Human *GAPDH*	AGAAGGCTGGGGCTCATTTG	AGGGGCCATCCACAGTCTTC

### ELISA analysis

The amounts of secreted IL-29, IL-6 and IL-8 in culture supernatants of MH7A cells were measured by ELISA according to the manufacturer’s instructions.

### Flow cytometry analysis

After incubation with IL-29, MH7A cells were pelleted by centrifugation at 450 *g* for 5 minutes, then fixed and permeabilized by using a cell fixation permeabilization kit. Briefly, thoroughly resuspended cells were added to 100 μl of BD Cytofix/Cytoperm solution and incubated for 20 minutes at 4°C. Cells were then incubated with FITC-conjugated rat anti-human TLR2, TLR3 and TLR4 monoclonal antibody or isotope control, respectively (at a final concentration 4 μg/ml), at 4°C for 30 minutes. After cells were washed, they were analyzed on a FACSCalibur flow cytometer (BD Biosciences, San Jose, CA, USA).

### Statistical analysis

Statistical analyses were performed with SPSS software (SPSS, Inc, Chicago, IL, USA), and all figures were drawn using GraphPad Prism 5.0 software (GraphPad Software, La Jolla, CA, USA). Data were expressed as means ± SD. Differences between two groups were calculated using Student’s *t*-test for parametric data and the Mann–Whitney *U* test for nonparametric data. For all experiments, *P* < 0.05 was taken as significant.

## Results

### Expression of IL-29 receptor in rheumatoid arthritis synovial fibroblasts

**Figure 1:**
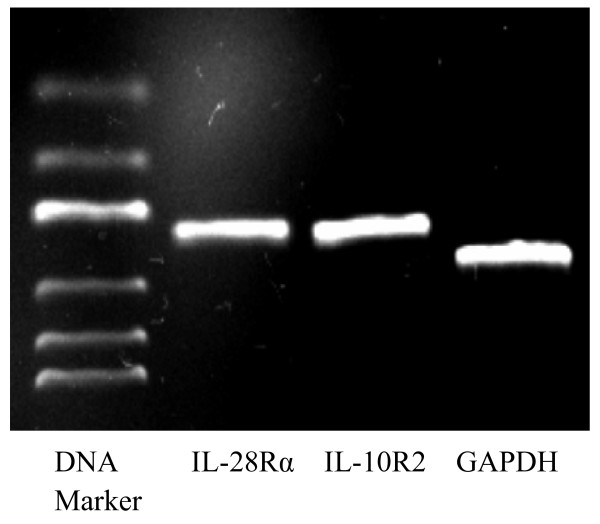
**IL-29 receptor IL-28Rα and IL-10R2 expression in rheumatoid arthritis synovial fibroblasts.** Expression of both receptor subunits (IL-28Rα and IL-10R2) was detected in MH7A cells using semiquantitative RT-PCR analysis. Representative PCR products after gel electrophoresis are shown. GAPDH, glyceraldehyde 3-phosphate dehydrogenase.

The biological functions of IL-29 depend mainly on its receptor, thus we first examined the expression of the IL-29 receptor complex, consisting of IL-28Rα and IL-10R2, in MH7A cells. The results showed that RA-FLS could express mRNA of both IL-28Rα and IL-10R2 in MH7A cells (Figure 1) and primary RA-FLS (see Additional file 2: Figure S1), implying that RA-FLS is able to respond to IL-29 stimulation.

### IL-29 induces protein expression of IL-6 and IL-8 in rheumatoid arthritis synovial fibroblasts

**Figure 2:**
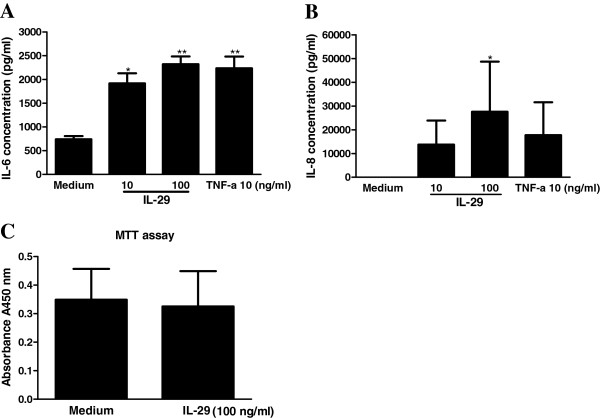
**IL-29 induces production of IL-6 and IL-8 by rheumatoid arthritis synovial fibroblasts.** MH7A cells were cultured with IL-29 (1, 10 and 100 ng/ml) or TNF-α (10 ng/ml; positive control) for 24 hours, and the production of IL-6 **(A)** and IL-8 **(B)** were measured by ELISA. Because the effects of IL-29 at 1 ng/ml on the production of IL-6 and IL-8 in MH7A cells are similar to the medium control group, the data from IL-29 at 1 ng/ml in MH7A are not shown. MH7A cells were also cultured in the presence or absence of IL-29 (100 ng/ml) for 24 hours, then cell viability was measured by 3-(4,5-dimethylthiazol-2-yl)-2,5-diphenyltetrazolium bromide assay **(C)**. The data present the mean ± SD for three independent experiments. *P < 0.05 vs medium control. **P < 0.01 vs medium control.

We next examined whether IL-29 induces IL-6 and IL-8 release from MH7A cells, as we previously reported that IL-29 upregulated IL-6 and IL-8 mRNA expression in MH7A cells [13]. Consistent with this information, the new data showed that protein levels of IL-6 and IL-8 were markedly increased in MH7A cells upon IL-29 stimulation in a dose-dependent manner (Figure 2). In addition, using an MTT assay, we found that IL-29 enhanced production of IL-6 and IL-8 without affecting the viability of MH7A cells (Figure 2C). We also observed that IL-29 induced IL-6 and IL-8 production in the primary culture of FLS from RA patients (see Additional file 3: Figure S2). Together, these data confirm that IL-29 triggers proinflammatory cytokine production in RA-FLS. To further examine the kinetic changes of IL-29-induced IL-6 and IL-8 production, we found that IL-6 and IL-8 production started to increase at 6 hours and reached a peak at 24 hours after IL-29 treatment (see Additional file 4: Figure S3).

### IL-29 enhances Toll-like receptor-mediated IL-6 and IL-8 expression in rheumatoid arthritis synovial fibroblasts

**Figure 3:**
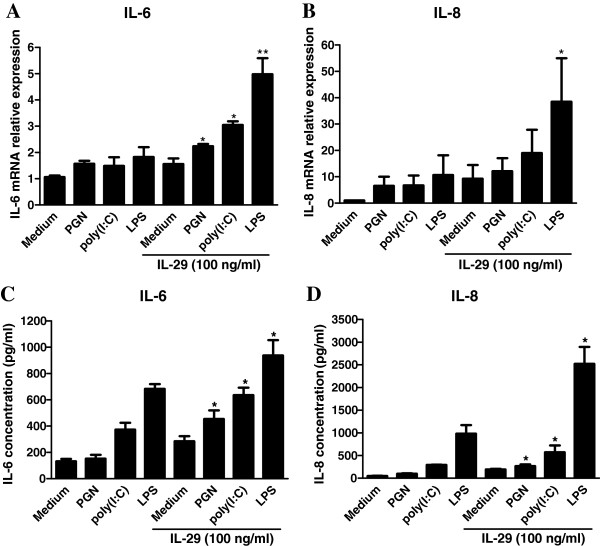
**IL-29 enhances Toll-like receptors 2-, 3- and 4-mediated IL-6 and IL-8 expression in MH7A cells.** MH7A cells were pretreated with IL-29 (100 ng/ml) for 24 hours, then further stimulated with peptidoglycan (PGN) (2.5 μg/ml), polyinosinic-polycytidylic acid (poly(I:C)) (25 μg/ml) or lipopolysaccharide (LPS) (100 ng/ml). Expression of mRNA **(A)** and **(B)** and protein **(C)** and **(D)** of IL-6 and IL-8 was determined by real-time PCR and ELISA, respectively. The results shown are representative of one of three independent experiments. The error bars represent mean ± SD for triplicate wells. *P < 0.05 vs medium control. **P < 0.01 vs medium control.

Because IL-29 and TLR2, TLR3 and TLR4 have all been reported to be highly expressed in RA synovium and to induce IL-6 and IL-8 expression in RA-FLSs, we further investigated the potential regulatory relationship between cytokines IL-29, IL-6 and IL-8 and TLR2, TLR3 and TLR4 in RA-FLSs. MH7A cells were pretreated with IL-29 (100 ng/ml), then further stimulated with PGN, poly(I:C) or LPS, respectively. We found that IL-29 increased IL-6 mRNA expression by approximately 1.4-, 2.0- and 2.7-fold, and IL-8 expression by 1.86-, 2.8- and 3.6-fold, after PGN, poly(I:C) or LPS stimulation, respectively (Figures 3A and 3B). In addition, IL-29 significantly enhanced IL-6 and IL-8 protein expression after PGN, poly(I:C) or LPS stimulation, respectively (Figure 3C and 3D). We further compared the difference in protein levels of IL-6 and IL-8 in MH7A cells pretreated with IL-29, then stimulated them with TLR ligands or cells preincubated with TLR ligands and subsequently stimulated them with IL-29. We did not find clear differences between the two settings in the stimulated cells (see Additional file 5: Figure S4). These data show that TLR2, TLR3 or TLR4 stimulation leads to production of IL-6 and IL-8, which was significantly enhanced when the cells were primed with IL-29 compared with cells stimulated with TLR ligand alone.

### IL-29 induces expression of Toll-like receptors 2, 3 and 4 in rheumatoid arthritis synovial fibroblasts

**Figure 4:**
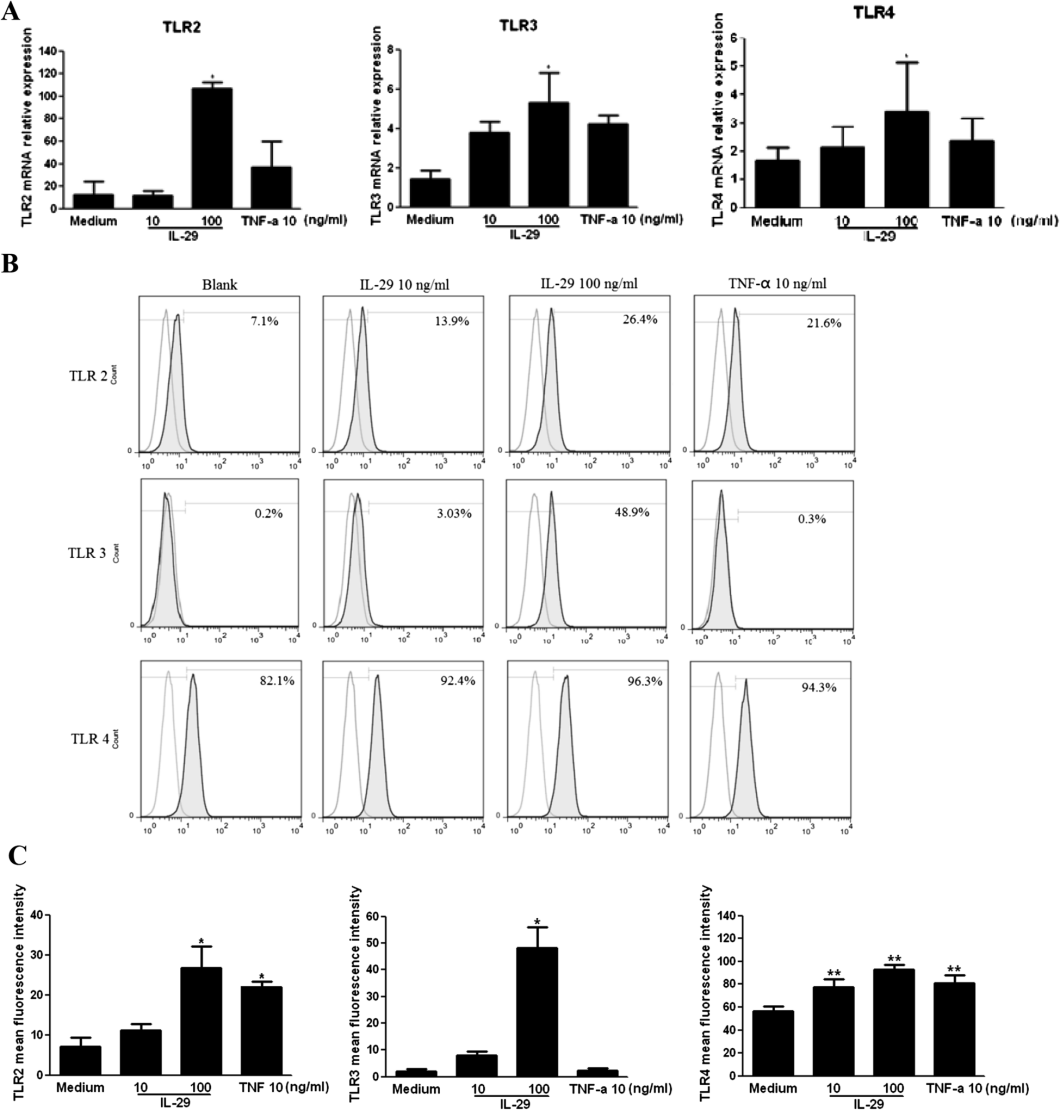
**IL-29 induces production of Toll-like receptors 2, 3 and 4 in MH7A cells.** MH7A cells were stimulated with IL-29 or TNF-α (positive control) for 24 hours, then the expression of Toll-like receptor 2 (*TLR2*), *TLR3* and *TLR4* mRNA **(A)** or protein **(B)** was measured by real-time PCR or flow cytometry analysis, respectively. **(C)** Quantitative expression of *TLR2*, *TLR3* and *TLR4* protein was summarized. Because the effect of IL-29 at 1 ng/ml on the expression of the above genes in MH7A cells is similar to the medium control group, the data from IL-29 at 1 ng/ml in MH7A are not shown in the figure. The data depicted in (A) and (B) are representative of one of three independent experiments. The error bars represent mean ± SD for triplicate wells. The data presented in (C) are the means ± SD for three independent experiments. *P < 0.05 versus medium control. **P < 0.01 versus medium control.

It has been reported that type I IFN induces TLR2, TLR3 and TLR4 expression in RA-FLSs; therefore, we examined whether IL-29 enhances TLR2-, TLR3- and TLR4-mediated IL-6 and IL-8 expression in RA-FLSs through increased TLR expression. The results showed that TLR2, TLR3 and TLR4 mRNA were upregulated in a dose-dependent manner and a 8.5-, 3.7- and 2.1-fold increases in TLR2, TLR3 and TLR4 mRNA expression, respectively, were observed in RA-FLSs after incubation with 100 ng/ml IL-29 for 24 hours (Figure 4A). Quantitative analysis of protein levels is summarized in Figure 4C. In line with the mRNA expression change, TLR2, TLR3 and TLR4 protein expression was also increased in a dose-dependent manner after IL-29 incubation. These data indicate that IL-29 enhances TLR expression in RA-FLSs. We also found that PGN, poly(I:C) or LPS showed a more robust capacity in inducing TLR2, TLR3 or TLR4 production compared with IL-29 treatment (100 ng/ml) (see Additional file 6: Figure S5).

## Discussion

In our previous study, we reported that IL-29 may contribute to synovial inflammation during RA pathogenesis. In this study, we further investigated the underlying molecular mechanism by which IL-29 contributes to RA inflammation. We examined whether IL-29 affects cytokine production in response to TLR ligands to determine its affect on the classical pattern recognition initiated by FLS-driven responses. Our results show for the first time that IL-29 is able to enhance TLR2, TLR3 and TLR4 expression in RA-FLS, leading to increased production of IL-6 and IL-8. This conclusion is supported by the following observations: (1) IL-29 induces IL-6 and IL-8 mRNA and protein expression in RA-FLS; (2) IL-29 increases mRNA and protein expression of TLR2, TLR3 and TLR4 in RA-FLS; and (3) TLR2, TLR3 and TLR4 ligands elevate the expression of IL-6 and IL-8 in RA-FLS.

IL-29 has been shown to activate monocytes and macrophages to produce a restricted panel of cytokines. Jordan *et al*. reported that IL-29 alone, in the absence of TLR ligands, induced high levels of IL-6 and IL-8 by human monocytes, which was further enhanced by LPS [8]. However, several other groups were unable to observe the induction of IL-6 and IL-8 by IL-29 in the absence of TLR ligands because no IL-29 receptor was unexpressed in human primary monocytes [25,27,28]. We previously reported that MH7A cells express IL-28Rα (the specific receptor of IL-29) evidenced by immunofluorescence staining [13]. In the present study, we show that the complex receptor of IL-29, including IL-28Rα and IL-10R2, is expressed in both human primary RA-FLSs and RA-FLS cell line MH7A cells. Therefore, RA-FLSs are responsive to IL-29 and may release IL-6 and IL-8 in the absence of TLR ligands. We also found the increased protein expression of IL-6 and IL-8 in RA-FLSs after IL-29 stimulation, which is in agreement with the change in mRNA level of IL-6 and IL-8 after IL-29 stimulation, as we reported in our previous study. Induction of IL-6 and IL-8 (an important chemoattractant for neutrophils) by IL-29 suggests a link between the innate and adaptive response and early recruitment of inflammatory cells to the site of inflammation by IL-29.

In addition to induction of IL-6 and IL-8 production by IL-29 in RA-FLSs, we also observed that IL-29 enhances TLR2, TLR3 and TLR4 mRNA and protein expression in RA-FLSs. This is important because TLR and its ligands are abundant in RA synovium, which is needed for the initiation of a chronic and persisting inflammatory response in RA. TLR2 and TLR4 elicit immune responses on binding of antigens from bacteria and endogenous ligands, leading to the production of inflammatory mediators. TLR3 serves as a receptor for viral nucleic acids and have a key role in antiviral immunity. To the best of our knowledge, upregulation of TLR2, TLR3 and TLR4 by IL-29 in RA-FLS has not been previously reported.

Taken together, our data provide valid evidence that IL-29 could trigger cytokines and TLRs expression in FLS cell line and primary cells. Considering that we previously reported that synovial macrophages and fibroblasts might be the source of the high expression of IL-29 in inflamed joint of RA, it is possible that high levels of IL-29 derived from either synovial macrophages or fibroblasts in turn contribute to RA pathogenesis.

In addition, the ligands of TLR2, TLR3 and TLR4 are reported to induce IL-29 production in dendritic cells and macrophages [29]. In line with this, our supplementary data also document that stimulation of TLR2, TLR3 and TLR4 leads to an increased production of IL-29 in RA-FLSs for the first time (see Additional file 7: Figure S6). Considering that macrophages produce IL-29 early in the course of viral infection [30], it is tempting to speculate that the induction of IL-29 is an early event following challenge with bacterially or virally derived pathogens. IL-29 subsequently activates RA-FLSs to release IL-6, IL-8 or other proinflammatory cytokines via TLR-mediated signaling and contributes to the chronic inflammation in RA. The exact mechanism underpinning the augmented TLR response after preincubation with IL-29 is not yet understood. On the basis of our data, we conclude that this phenomenon results from increased expression of TLR2, TLR3 and TLR4.

## Conclusions

In this study, we have demonstrated the involvement of IL-29 in an augmented TLR2, TLR3 and TLR4-mediated inflammatory cytokine production. Our data imply an important role of IL-29 in the activation and sustained synovial inflammation of RA.

## Abbreviations

DMEM: Dulbecco’s modified Eagle’s medium; ELISA: Enzyme-linked immunosorbent assay; FBS: Fetal bovine serum; FLS: Synovial fibroblast; IFN: Interferon; IL: Interleukin; PCR: Polymerase chain reaction; RA: Rheumatoid arthritis; RA- TLR: Toll-like receptor; TNF-α: Tumor necrosis factor α.

## Competing interests

The authors declare that they have no competing interests.

## Authors’ contributions

LX, XF, WG and DG performed experiments. FW, WT and MZ contributed to drafting the manuscript and to study design and data analysis. All authors read and approved the final manuscript.

## Supplementary Material

Additional file 1 **Primary RA fibroblasts isolation and culture.** Primary RA fibroblasts (RA-FLS) were isolated by enzymatic digestion of synovial tissues obtained from RA patients undergoing total knee replacement surgery. In general, synovial tissue was minced and digested with 1% collagenase II at 37°C for 4 h. RA-FLS were cultured in DMEM medium supplemented with 10% fetal bovine serum (FBS), 100 U/ml penicillin and 100 μg/ml streptomycin at 37°C in a humidified atmosphere of 5% CO2 in air. This study was approved by the Ethical Committee of the First Affiliated Hospital of Nanjing Medical University, and informed consent was obtained from all patients. Click here for file

Additional file 2: Figure S1 IL-29 receptor IL-28Rα and IL-10R2 expression in primary RA-FLS. Expression of IL-29 receptor subunits IL-28Rα and IL-10R2 in primary RA-FLS was detected by semi-quantitative RT-PCR analysis. Representative gel electrophoresis of PCR products were shown in the figure. Click here for file

Additional file 3: Figure S2 IL-29 induces protein expression of IL-6 and IL-8 in primary RA-FLS. The primary RA-FLS were cultured with IL-29 (1, 10, 100 ng/ml) or TNF-a (10 ng/ml, a positive control) for 24 h and the production of IL-6 (A&D) and IL-8 (B& E) were measured using ELISA. Because the effects of IL-29 at 1 ng/ml on the production of IL-6, 8 in primary RA-FLS are similar to the medium control group, the data from IL-29 at 1 ng/ml in primary RA-FLS are not shown in the figure. The data present the mean ± SD for 3 independent experiments. **p < 0.01 versus medium control. Click here for file

Additional file 4: Figure S3 IL-29 induces IL-6 and IL-8 gene and protein expression at different time points. MH7A cells were stimulated with IL-29 (100 ng/ml) for 6 h, 12 h or 24 h, and then the expression of IL-6 and IL-8 mRNA (A & B) or protein (C & D) was measured by real time PCR or ELISA analysis, respectively. The data show representative of one out three independent experiments. The error bars represent mean ± SD for triplicate wells. *p < 0.05 versus medium control. Click here for file

Additional file 5: Figure S4 IL-29 shows similar effect on TLR2, 3, 4-mediated IL-6, IL-8 production after two different treatments in MH7A cells. MH7A cells were pretreated with IL-29 (100 ng/ml) for 24 h, and then further stimulated with PGN (2.5 μg/ml), poly I:C (25 μg/ml) or LPS (100 ng/ml). In contrast, MH7A cells were pretreated with PGN (2.5 μg/ml), poly I:C (25 μg/ml) or LPS (100 ng/ml) for 24 h, and then further stimulated with IL-29 (100 ng/ml) for 24 h. The concentration of IL-6 (A) and IL-8 (B) in cell supernatant was examined with ELISA. The data present the mean ± SD for 3 independent experiments. *p < 0.05 versus medium control. **p < 0.01 versus medium control. Click here for file

Additional file 6: Figure S5 IL-29 induces production of TLR2, 3 and 4 in MH7A cells. MH7A cells were stimulated with IL-29 (100 ng/ml) or PGN (2.5 μg/ml), poly I:C (25 μg/ml) or LPS (100 ng/ml) (a positive control for TLR2, 3 and 4, respectively) for 24 h, and then the expression of TLR2, 3 and 4 mRNA (A) or protein (B) was measured by real time PCR or flow cytometric analysis, respectively. (C) Quantitative expression of TLR2, 3 and 4 protein was summarized. The data depicted in (A) and (B) show representative of one out three independent experiments. The error bars represent mean ± SD for triplicate wells. The data present in (C) show the means ± SD for 3 independent experiments. *p < 0.05 versus medium control. **p < 0.01 versus medium control. Click here for file

Additional file 7: Figure S6 TLR ligands induce IL-29 expression in RA-FLS. MH7A cells or primary RA-FLS were stimulated with PGN (2.5 μg/ml), poly I:C (25 μg/ml) or LPS (100 ng/ml) alone for 24 h. The total cellular RNA was isolated for cDNA synthesis and real time PCR was performed to determine the relative expression of IL-29 mRNA (A & C). The values were normalized against GAPDH mRNA and relative gene expression was determined by the 2^-∆∆ct^ method. The data show representative of one out three independent experiments. The error bars represent mean ± SD for triplicate wells. (B & D) Cell culture supernatants were harvested and analyzed by ELISA kits. The data represent the mean ± SD of 3 separate experiments. *p < 0.05 versus medium control. Click here for file
