# Supplementary material for: IL-29 enhances Toll-like receptor-mediated IL-6 and IL-8 production by the synovial fibroblasts from rheumatoid arthritis patients
Source: Arthritis Res Ther. 2013 Oct 29;15(5):R170. doi: 10.1186/ar4357 (PMC3978693; doi:10.1186/ar4357)
Supplement: Additional file 1 — Primary RA fibroblasts isolation and culture. Primary RA fibroblasts (RA-FLS) were isolated by enzymatic digestion of synovial tissues obtained from RA patients undergoing total knee replacement surgery. In general, synovial tissue was minced and digested with 1% collagenase II at 37°C for 4 h. RA-FLS were cultured in DMEM medium supplemented with 10% fetal bovine serum (FBS), 100 U/ml penicillin and 100 μg/ml streptomycin at 37°C in a humidified atmosphere of 5% CO2 in air. This study was approved by the Ethical Committee of the First Affiliated Hospital of Nanjing Medical University, and informed consent was obtained from all patients. [file ar4357-S1.doc]

**Methods**

**Primary RA fibroblasts isolation and culture**

Primary RA fibroblasts (RA-FLS) were isolated by enzymatic digestion of synovial tissues obtained from RA patients undergoing total knee replacement surgery. In general, synovial tissue was minced and digested with 1% collagenase II at 37℃ for 4 h. RA-FLS were cultured in DMEM medium supplemented with 10% fetal bovine serum (FBS), 100 U/ml penicillin and 100 µg/ml streptomycin at 37℃ in a humidified atmosphere of 5% CO_2_ in air. This study was approved by the Ethical Committee of the First Affiliated Hospital of Nanjing Medical University, and informed consent was obtained from all patients.
