# Supplementary figures and images for: IL-29 enhances Toll-like receptor-mediated IL-6 and IL-8 production by the synovial fibroblasts from rheumatoid arthritis patients
Source: Arthritis Res Ther. 2013 Oct 29;15(5):R170. doi: 10.1186/ar4357 (PMC3978693; doi:10.1186/ar4357)

**Figure S1**

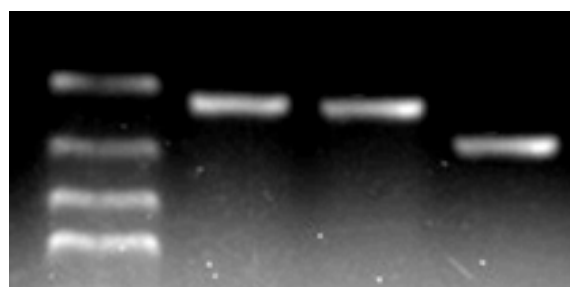

Marker IL-28R $\alpha$  IL-10R2 GAPDH

Supplement: Additional file 2: Figure S1 — IL-29 receptor IL-28Rα and IL-10R2 expression in primary RA-FLS. Expression of IL-29 receptor subunits IL-28Rα and IL-10R2 in primary RA-FLS was detected by semi-quantitative RT-PCR analysis. Representative gel electrophoresis of PCR products were shown in the figure. [file ar4357-S2.pdf]

Figure S2

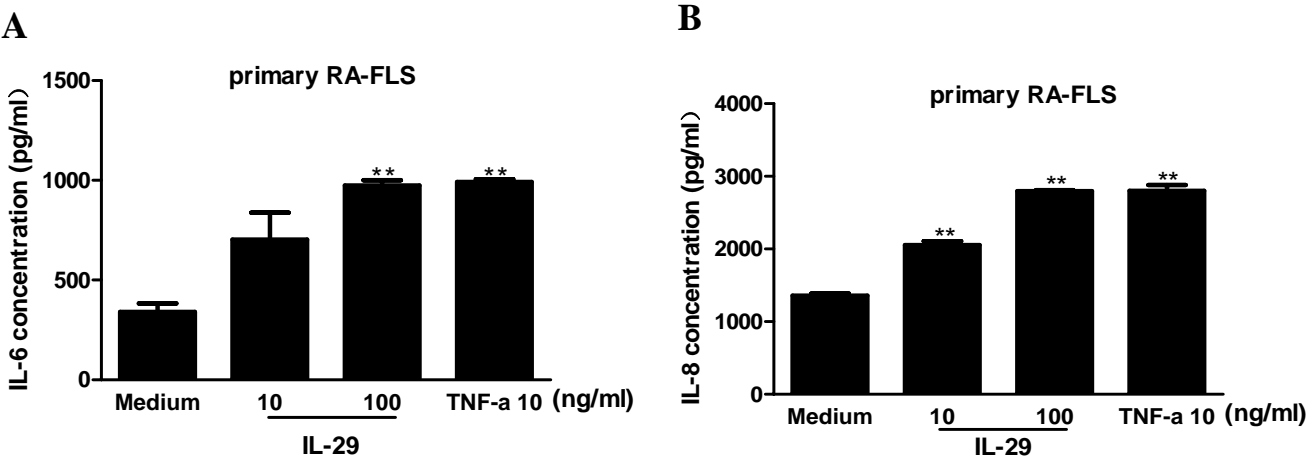

Supplement: Additional file 3: Figure S2 — IL-29 induces protein expression of IL-6 and IL-8 in primary RA-FLS. The primary RA-FLS were cultured with IL-29 (1, 10, 100 ng/ml) or TNF-a (10 ng/ml, a positive control) for 24 h and the production of IL-6 (A&D) and IL-8 (B& E) were measured using ELISA. Because the effects of IL-29 at 1 ng/ml on the production of IL-6, 8 in primary RA-FLS are similar to the medium control group, the data from IL-29 at 1 ng/ml in primary RA-FLS are not shown in the figure. The data present the mean ± SD for 3 independent experiments. **p < 0.01 versus medium control. [file ar4357-S3.pdf]

Figure S3

A

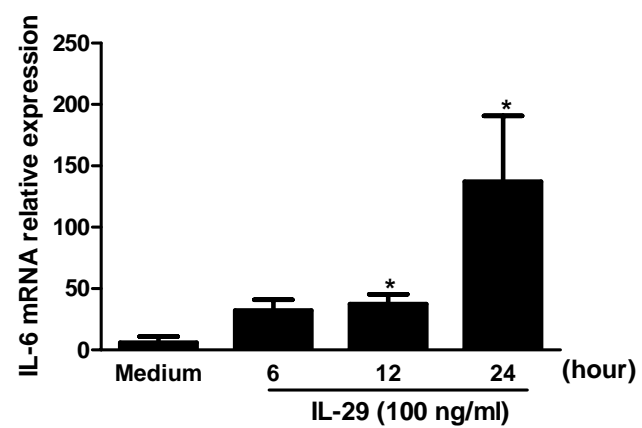

B

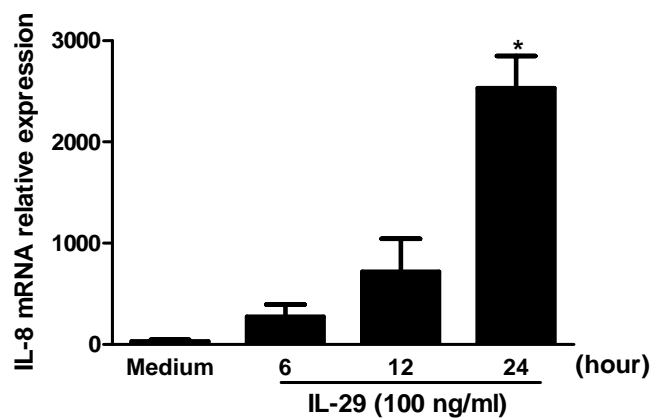

C

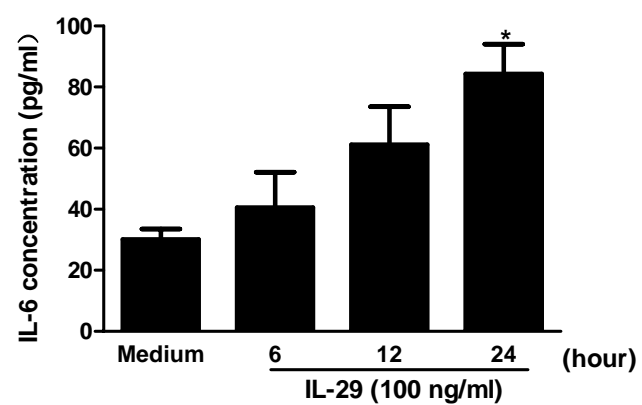

D

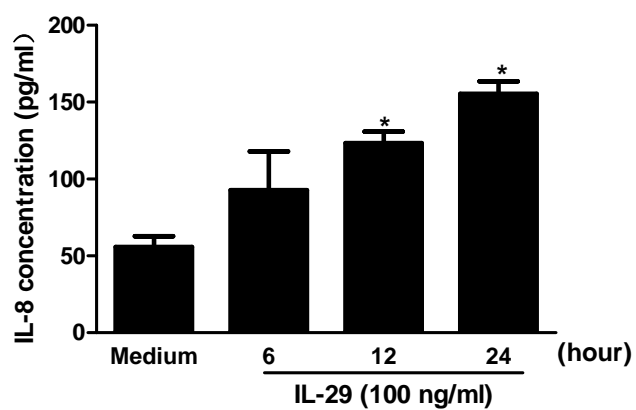

Supplement: Additional file 4: Figure S3 — IL-29 induces IL-6 and IL-8 gene and protein expression at different time points. MH7A cells were stimulated with IL-29 (100 ng/ml) for 6 h, 12 h or 24 h, and then the expression of IL-6 and IL-8 mRNA (A & B) or protein (C & D) was measured by real time PCR or ELISA analysis, respectively. The data show representative of one out three independent experiments. The error bars represent mean ± SD for triplicate wells. *p < 0.05 versus medium control. [file ar4357-S4.pdf]

Figure S4

A

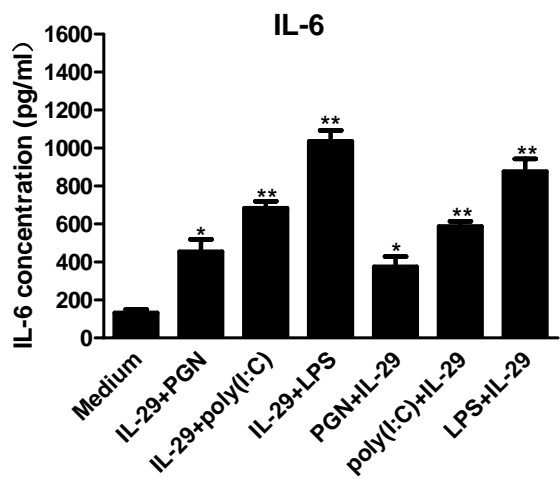

B

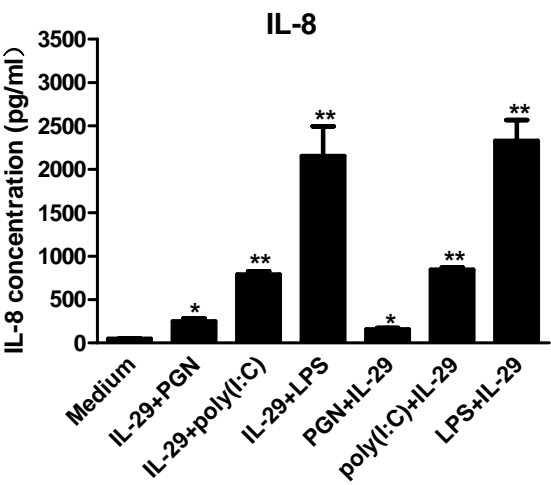

Supplement: Additional file 5: Figure S4 — IL-29 shows similar effect on TLR2, 3, 4-mediated IL-6, IL-8 production after two different treatments in MH7A cells. MH7A cells were pretreated with IL-29 (100 ng/ml) for 24 h, and then further stimulated with PGN (2.5 μg/ml), poly I:C (25 μg/ml) or LPS (100 ng/ml). In contrast, MH7A cells were pretreated with PGN (2.5 μg/ml), poly I:C (25 μg/ml) or LPS (100 ng/ml) for 24 h, and then further stimulated with IL-29 (100 ng/ml) for 24 h. The concentration of IL-6 (A) and IL-8 (B) in cell supernatant was examined with ELISA. The data present the mean ± SD for 3 independent experiments. *p < 0.05 versus medium control. **p < 0.01 versus medium control. [file ar4357-S5.pdf]

Figure S5

A

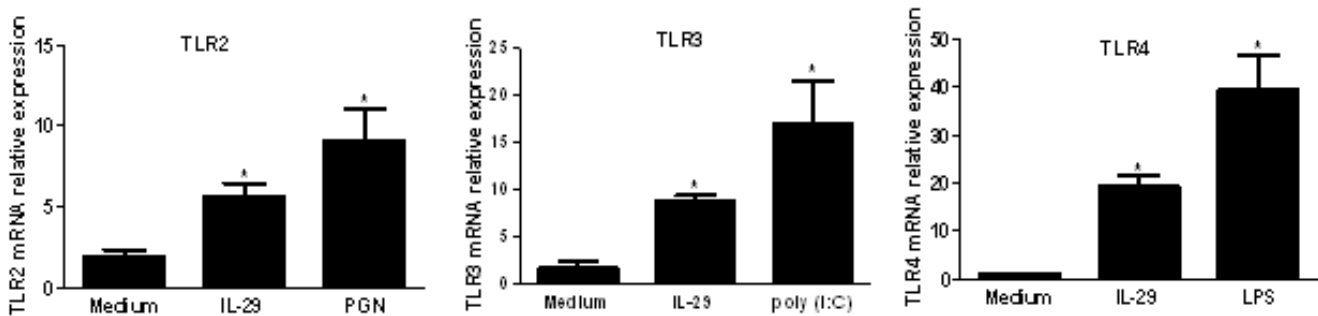

B

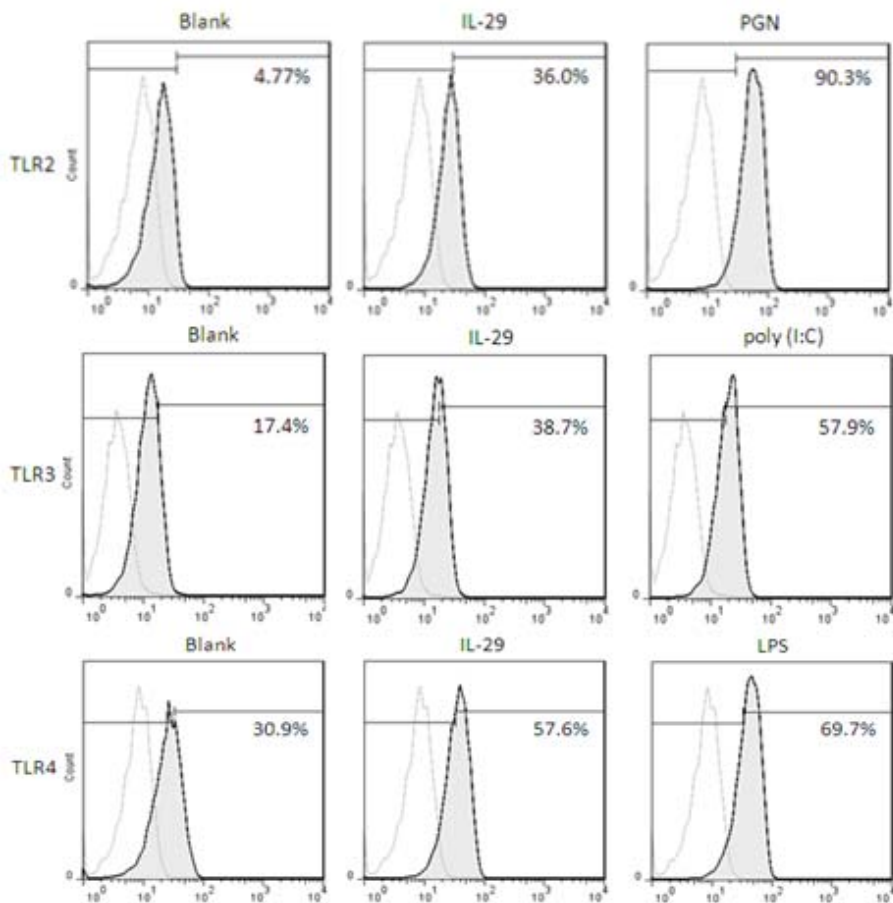

C

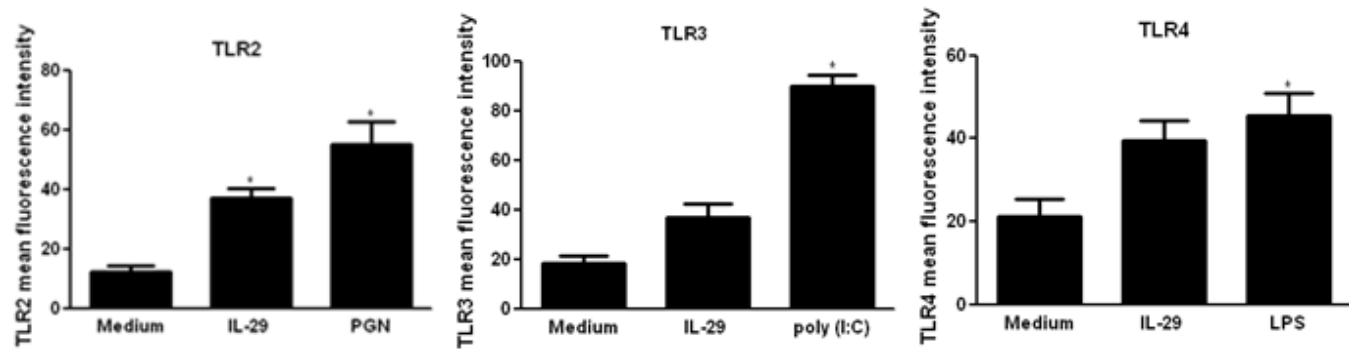

Supplement: Additional file 6: Figure S5 — IL-29 induces production of TLR2, 3 and 4 in MH7A cells. MH7A cells were stimulated with IL-29 (100 ng/ml) or PGN (2.5 μg/ml), poly I:C (25 μg/ml) or LPS (100 ng/ml) (a positive control for TLR2, 3 and 4, respectively) for 24 h, and then the expression of TLR2, 3 and 4 mRNA (A) or protein (B) was measured by real time PCR or flow cytometric analysis, respectively. (C) Quantitative expression of TLR2, 3 and 4 protein was summarized. The data depicted in (A) and (B) show representative of one out three independent experiments. The error bars represent mean ± SD for triplicate wells. The data present in (C) show the means ± SD for 3 independent experiments. *p < 0.05 versus medium control. **p < 0.01 versus medium control. [file ar4357-S6.pdf]

Figure S6

A

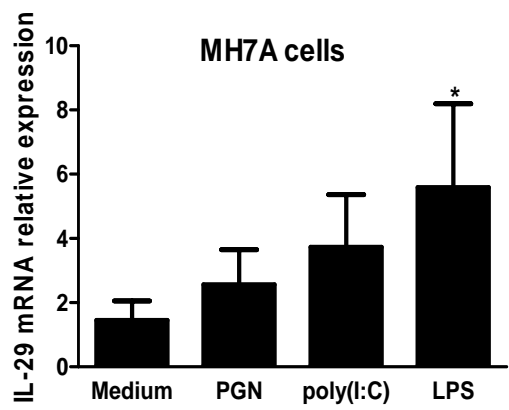

B

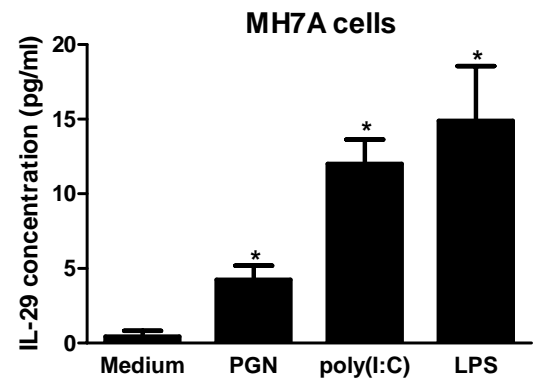

C

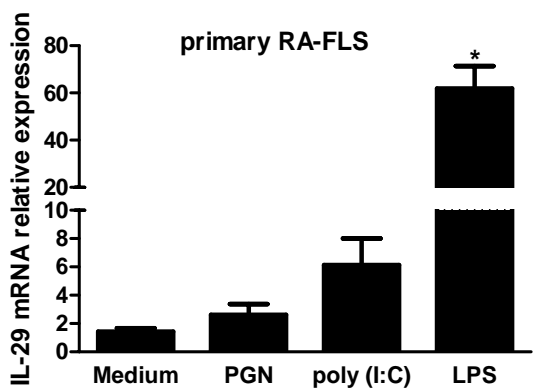

D

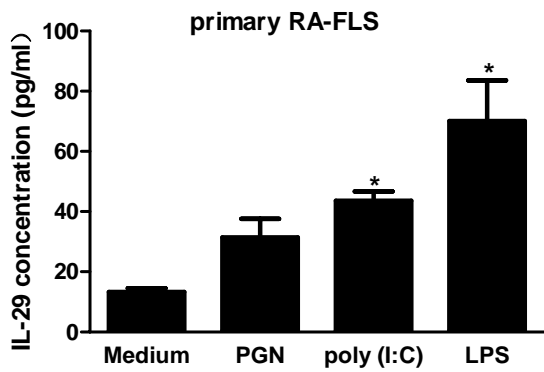

Supplement: Additional file 7: Figure S6 — TLR ligands induce IL-29 expression in RA-FLS. MH7A cells or primary RA-FLS were stimulated with PGN (2.5 μg/ml), poly I:C (25 μg/ml) or LPS (100 ng/ml) alone for 24 h. The total cellular RNA was isolated for cDNA synthesis and real time PCR was performed to determine the relative expression of IL-29 mRNA (A & C). The values were normalized against GAPDH mRNA and relative gene expression was determined by the 2-∆∆ct method. The data show representative of one out three independent experiments. The error bars represent mean ± SD for triplicate wells. (B & D) Cell culture supernatants were harvested and analyzed by ELISA kits. The data represent the mean ± SD of 3 separate experiments. *p < 0.05 versus medium control. [file ar4357-S7.pdf]
